# Antimicrobial Resistance Genes in ESBL-Producing *Escherichia coli* Isolates from Animals in Greece

**DOI:** 10.3390/antibiotics10040389

**Published:** 2021-04-04

**Authors:** Zoi Athanasakopoulou, Martin Reinicke, Celia Diezel, Marina Sofia, Dimitris C. Chatzopoulos, Sascha D. Braun, Annett Reissig, Vassiliki Spyrou, Stefan Monecke, Ralf Ehricht, Katerina Tsilipounidaki, Alexios Giannakopoulos, Efthymia Petinaki, Charalambos Billinis

**Affiliations:** 1 Faculty of Veterinary Science, University of Thessaly, 43100 Karditsa, Greece; zathanas@uth.gr (Z.A.); msofia@uth.gr (M.S.); dchatzopoulos@uth.gr (D.C.C.); algiannak@uth.gr (A.G.); 2 Leibniz Institute of Photonic Technology (IPHT), 07745 Jena, Germany; martin.reinicke@leibniz-ipht.de (M.R.); Celia.diezel@leibniz-ipht.de (C.D.); sascha.braun@leibniz-ipht.de (S.D.B.); annett.reissig@leibniz-ipht.de (A.R.); stefan.monecke@leibniz-ipht.de (S.M.); Ralf.Ehricht@leibniz-ipht.de (R.E.); 3 InfectoGnostics Research Campus, 07743 Jena, Germany; 4 Faculty of Animal Science, University of Thessaly, 41110 Larissa, Greece; vasilikispyrou@uth.gr; 5 Institut fuer Medizinische Mikrobiologie und Hygiene, Medizinische Fakultaet “Carl Gustav Carus”, TU Dresden, 01307 Dresden, Germany; 6 Institute of Physical Chemistry, Friedrich Schiller University Jena, 07737 Jena, Germany; 7 Faculty of Medicine, University of Thessaly, 41500 Larissa, Greece; tsilipou@uth.gr (K.T.); petinaki@uth.gr (E.P.); 8 Faculty of Public and Integrated Health, University of Thessaly, 43100 Karditsa, Greece

**Keywords:** ESBL, *Escherichia coli*, multidrug resistance, antimicrobial resistance genes, cattle, pigs, Eurasian magpie, Greece

## Abstract

The prevalence of multidrug resistant, extended spectrum β-lactamase (ESBL)-producing Enterobacteriaceae is increasing worldwide. The present study aimed to provide an overview of the multidrug resistance phenotype and genotype of ESBL-producing *Escherichia coli* (*E. coli*) isolates of livestock and wild bird origin in Greece. Nineteen phenotypically confirmed ESBL-producing *E. coli* strains isolated from fecal samples of cattle (*n* = 7), pigs (*n* = 11) and a Eurasian magpie that presented resistance to at least one class of non β-lactam antibiotics, were selected and genotypically characterized. A DNA-microarray based assay was used, which allows the detection of various genes associated with antimicrobial resistance. All isolates harbored *bla*_CTX-M-1/15_, while *bla*_TEM_ was co-detected in 13 of them. The AmpC gene *bla*_MIR_ was additionally detected in one strain. Resistance genes were also reported for aminoglycosides in all 19 isolates, for quinolones in 6, for sulfonamides in 17, for trimethoprim in 14, and for macrolides in 8. The *intI1* and/or tnpISE*cp1* genes, associated with mobile genetic elements, were identified in all but two isolates. This report describes the first detection of multidrug resistance genes among ESBL-producing *E. coli* strains retrieved from feces of cattle, pigs, and a wild bird in Greece, underlining their dissemination in diverse ecosystems and emphasizing the need for a One-Health approach when addressing the issue of antimicrobial resistance.

## 1. Introduction

The emergence and dissemination of extended-spectrum β-lactamase (ESBL) producing bacteria currently constitutes a major public health concern. ESBLs are enzymes that hydrolyze penicillins, first to third generation cephalosporins as well as aztreonam, at a rate that exceeds 10% of their hydrolysis rate for benzylpenicillin. They are inhibited by β-lactamase inhibitors such as clavulanic acid and utilize serine for β-lactam hydrolysis [1,2]. Over 9000 human deaths were caused by ESBL-producing Enterobacteriaceae in the USA in 2017 [3]. The same year, World Health Organization (WHO) ranked these resistant bacteria in the first priority tier, under the characterization ‘critical’, to guide research, discovery and development of new antibiotics [4].

ESBLs are divided into eleven families based on their amino acid sequences [5], with the CTX-M family, and particularly CTX-M-15 variant, currently predominating among ESBL-producing *Escherichia coli* (*E. coli*) strains [6]. Plasmidic location of ESBLs has been associated with multidrug resistance. Co-occurrence, on the same plasmid, of resistance determinants for cephalosporins, aminoglycosides, tetracycline, sulfonamides, carbapenems, and quinolones has been reported and is speculated to provide ESBL genes an advantage for maintenance due to co-selection processes [7,8]. Such plasmids also carry toxin/antitoxin systems that enforce maintenance, even in the absence of antimicrobial selective pressure [9]. These facts, combined with the bacterial ability for acquisition of multiple plasmids, has resulted in multiresistance among ESBL-producing strains, limiting the already few treatment options against these pathogens even further [10]. Mobile genetic elements—including insertion sequences, integrons, and transposons—have also significantly facilitated mobilization of *bla*_CTX-M_ onto different types of plasmids which assist the spread of ESBLs to a wide variety of hosts [11], rendering ESBL-producing *E. coli* an issue of great zoonotic importance.

The prevalence of presumptive ESBL-producing *E. coli* in the European Union, during 2017–2018, was reported to be 38% in fattening pigs and 25% in calves [12]. The therapeutic, metaphylactic and prophylactic use of antibiotics in veterinary medicine is considered to be the main cause for the selection of resistant bacteria in cattle and pigs, which are identified as a major ESBL reservoir [13]. However, ESBLs are also detected in Enterobacteriaceae isolated from hosts that do not consume antibiotics, i.e., wild fauna [14,15,16,17,18]. Wild birds are the most frequent ESBL carriers among wildlife species and have been proposed as another potential reservoir that can significantly contribute to the diffusion of resistant strains via migration and/or living in close proximity to both humans and other animals [19,20,21]. Notably, many of these wild birds are scavengers, including corvids [22], gulls, kites, vultures, storks [23], and cattle egrets [21].

In Greece, recently published data indicate overconsumption of antibiotics, as well as rates of antibiotic resistance consistently higher than in other EU member states [24,25,26]. Although ESBL-producing bacteria are frequently detected among humans, reports about animal isolates are scarce [27,28,29,30,31,32]. Both SHV and CTX-M types seem to be common in human strains [33,34], while mainly CTX-M variants have been reported from cattle, poultry, and dogs [30,31,32]. Human ESBL isolates have been detected to co-harbor various other resistance genes, such as plasmid mediated quinolone resistance genes (PMQR), carbapenemase genes and plasmid encoded AmpC genes [35,36,37], whereas animal ESBL isolates have only been correlated with a colistin resistance gene (*mcr-1*) [31].

To get a better insight into the characteristics of multidrug resistant ESBL producers of animal origin in Greece, the present study investigated the antimicrobial resistance profile of selected ESBL *E. coli* isolates from cattle, pigs and a wild bird. This is the first report describing the presence and presenting the multidrug resistance determinants of ESBL-producing strains in fecal samples of cattle, pigs, and wild birds in Greece.

## 2. Results

### 2.1. Phenotypic Antimicrobial Resistance of the ESBL-Producing E. coli

The 19 selected *E. coli* isolates from cattle (*n* = 7), pigs (*n* = 11) and a Eurasian magpie (*Pica pica*) presented resistance to penicillins (ampicillin), third (cefoperazone, ceftiofur), and fourth (cefquinome) generation cephalosporins, while they were susceptible to carbapenems. 

Among the seven cattle *E. coli* isolates, the ESBL phenotype was combined with aminoglycoside, fluoroquinolone, tetracycline, and trimethoprim/sulfamethoxazole resistance in four strains, with aminoglycoside, tetracycline, and trimethoprim/sulfamethoxazole resistance in two strains and with aminoglycoside and tetracycline resistance in one strain. 

The ESBL phenotype of pig isolates coexisted with resistance to aminoglycosides, fluoroquinolones, tetracycline, and trimethoprim/sulfamethoxazole in four strains; aminoglycoside, fluoroquinolone, and tetracycline resistance in one strain; tetracycline and trimethoprim/sulfamethoxazole resistance in four strains; fluoroquinolone resistance in one strain; and only tetracycline resistance in one strain.

The *E. coli* strain from the magpie presented the ESBL phenotype combined with reduced susceptibility to fluoroquinolones, tetracycline, and trimethoprim/sulfamethoxazole.

The antimicrobial resistance phenotype of each ESBL-producing *E. coli* isolate is summarized in Table 1.

### 2.2. Genotype of the ESBL-Producing E. coli

Microarray analysis confirmed that the 19 ESBL-producing strains belonged to *E. coli*. The genotyping results are presented in Table 1.

All seven bovine strains harbored *bla*_CTX-M-1/15_ and *bla*_TEM_, while the AmpC variant *bla*_MIR_ was identified, though not expressed, in only one strain. Carbapenemase or other β-lactamase genes were not detected in isolates of this animal species.

Furthermore, the presence of *bla*_CTX-M-1/15_ was confirmed in all 11 swine isolates, while six co-harbored *bla*_TEM_. AmpCs were not identified. A carbapenemase gene associated with the *bla*_OXA-134_ family was detected in one of the swine strains, namely S7-1. However, the corresponding phenotype, a resistance against imipenem, could not be detected (Table 1). Sequencing did not confirm the presence of a *bla*_OXA_ variant in this strain. The genes detected, using whole genome sequencing of the isolate S7-1, as well as their locations are presented in Supplementary Materials File S1.

The magpie’s isolate also harbored *bla*_CTX-M-1/15_ but no other β-lactamase variants were reported.

Regarding the seven cattle strains, all presented phenotypic resistance to aminoglycosides. The *aphA* resistance gene was detected in all seven, the *aadA1* in five, the *aadA2* in three, while *strA* and *strB* co-existed in six isolates. The *aadA1, aadA2*, *aphA*, *strA*, and *strB* gene pattern was reported in three and the *aphA*, *strA*, and *strB* in two strains. Reduced susceptibility to aminoglycosides was also detected in five pig isolates despite the fact that resistance genes were identified in all 11. *aadA1* was identified in eight strains, *aadA2* in six, *aadA4* in five, *aphA* in two and both *strA*, and *strB* in three strains. *strA* and *strB* were always concurrently detected. Co-occurrence of *aadA1*, *aadA2*, and *aadA4* was reported in three and of *aadA1* and *aadA4* in two strains. Finally, the wild bird’s isolate harbored *aadA4*, *strA*, and *strB*, without displaying phenotypic resistance.

*E. coli* strains were additionally tested for the presence of genes conferring resistance to quinolones. Four of the seven bovine isolates expressed a resistant phenotype, however only one harbored a PMQR gene, namely *qnrS*. Diminished susceptibility to this class of antibiotics was also identified in six of the 11 swine isolates, whereas resistance genes were detected in four. *qnrB* was identified in one, *qnrS* in two and co-occurrence of *qnrB* and *qnrS* was reported in one isolate. One of the swine strains that harbored *qnrS* (Table 1, isolate S7-2) did not present resistance to any of the fluoroquinolones tested. The wild bird’s isolate expressed a resistant phenotype and carried *qnrS*.

All seven cattle isolates harbored a minimum of one sulfonamide resistance gene. Specifically, *sul1* was identified in five strains, *sul2* in six, and *sul3* in one. Co-existence of *sul1* and *sul2* was reported in three isolates; and coexistence of *sul1*, *sul2*, and *sul3* was reported in one. Moreover, 9 of the 11 pig isolates presented at least one gene. s*ul1* was detected in six isolates, *sul2* in seven, and *sul3* in four. Co-occurrence of *sul1* and *sul2* was reported in three strains; of *sul2* and *sul3* in one; and of *sul1*, *sul2*, and *sul3* in two strains. The wild bird’s isolate harbored both *sul1* and *sul2*.

Genes associated with trimethoprim resistance were identified in five of the seven cattle isolates. *dfrA1* was detected in five and *dfrA5* in one. Coexistence of *dfrA1* and *dfrA5* was reported in one of the isolates. Concerning pig strains, eight out of the 11 carried at least one gene and a variety of genes were identified. Each one of *dfrA1*, *dfrA7*, *dfrA19* and *dfrA17* was found in five isolates, *dfrA12* in four, both *dfrA14* and *dfrA15* in two and *dfrA5* in one. Concurrent presence of *dfrA1*, *dfrA7*, *dfrA17*, and *dfrA19* was reported in four strains. The *dfrA7*, *dfrA17*, and *dfrA19* pattern was detected in one pig strain and in the isolate from the Eurasian magpie.

Overall, diminished susceptibility to sulfonamides/trimethoprim was expressed in all seven cattle, 8 of the 11 pig and in the wild bird isolates.

The *mph* gene, associated with macrolide resistance, was identified in one of the seven bovine strains, while 6 of the 11 swine as well as the magpie isolates harbored both *mph* and *mrx*.

### 2.3. Mobile Genetic Elements

Genes associated with mobile genetic elements were identified in all the seven bovine isolates. In detail, *intI1* was detected in five and tnpISE*cp1* in two strains. A total of 9 of the 11 swine strains harbored either *intI1* (*n* = 5) or tnpISE*cp1* (*n* = 1) or both (*n* = 3). *intI1* was also detected in the magpie’s isolate.

## 3. Discussion

The present study reports the antimicrobial resistance profile of 19 ESBL-producing *E. coli* strains isolated from cattle (*n* = 7), pigs (*n* = 11) and a Eurasian magpie in Greece. The genotypic antimicrobial resistance characteristics of the isolates were investigated by assessing the occurrence of a variety of resistance genes corresponding to seven classes of antibiotics. This is the first report presenting in detail the multidrug resistance determinants of ESBL-producing *E. coli* isolates of animal origin in Greece.

The reported ESBL-producing *E. coli* isolates presented diminished susceptibility to at least one agent of more than three classes of antibiotics and subsequently were characterized as multidrug resistant (MDR). The bovine strains were resistant to at least four classes of antibiotics, the swine strains expressed resistance to at least three antimicrobial classes and the magpie isolate displayed reduced susceptibility to five classes. Recent studies have confirmed the presence of MDR ESBL-producing *E. coli* among cattle [38,39], pigs [39,40], and wild birds [41,42], in various regions.

All the isolates expressed the ESBL phenotype due to *bla*_CTX-M-1/15_ carriage. CTX-M-1/15 are the most prevalent ESBL variants among humans, livestock, wild birds and the environment in Europe [19,43,44,45,46]. Several hospital and community-acquired infection outbreaks worldwide have been attributed to these enzymes, even in countries with low antibiotic consumption and low prevalence of antimicrobial resistance, such as Norway [47,48,49,50]. High ESBL occurrence among human isolates has been described in Greece and international travel to the country has been suggested as a significant risk factor for ESBL colonization [51,52]. However, there are no available data about the presence and molecular characteristics of ESBL-producing strains from livestock and wildlife. To the best of our knowledge, this is the first identification of *bla*_CTX-M-1/15_ in fecal *E. coli* isolates of cattle, pigs, and a wild bird in Greece. Detection of these variants among strains from the abovementioned species is alarming and probably depicts their wide dissemination, since they were retrieved from different ecological niches, i.e., farmed animals and wildlife. These genes have also formerly been detected in isolates from healthy dogs in Greece [30]. We did not detect *bla*_SHV_ variants, although in our country they have previously been identified in milk samples from cows presenting mastitis and are frequently reported among human strains [31,33].

The *bla*_TEM_ β-lactamase gene was identified in 13 of the 19 ESBL isolates; all the cattle and six swine isolates. However, we cannot assume whether these genes encoded ESBLs or the narrow spectrum β-lactamases TEM-1 or TEM-2 since the array includes consensus probes and the TEM subtype was not identified. *bla*_MIR_ was the only plasmidic AmpC gene detected in one cattle isolate which was, though, not resistant to β-lactams/β-lactamase inhibitor combinations [53]. Additionally, a carbapenemase gene associated with the family *bla*_OXA-134_ was detected in a pig strain. Since signals on the microarray assay were weak, a new allelic variant of this gene/gene-family was suspected. However, subsequent sequencing did not confirm the presence of an OXA-134-like gene. Reviewing published data, genes of this family have only been isolated from *Acinetobacter spp.*, which is their natural host [54,55], and do not constitute a clinical problem to date.

Genes associated with aminoglycoside resistance were detected in all the *E. coli* isolates, although only 12 expressed a resistant phenotype. Aminoglycosides are extensively used in veterinary medicine [56], a fact that could explain the wide dissemination of the respective resistance genes. Recent studies that molecularly characterized ESBL-producing strains, confirmed a high frequency of aminoglycoside resistance genes among isolates from pigs and abattoir workers in Cameroon [57] as well as from retail raw pork and beef meat in Singapore [58]. According to our results, *aadA1*, *aphA*, *strA*, and *strB* were the most frequently detected genes in bovine strains, as has recently been reported in ESBL-producing *E. coli* isolated from milk samples of cattle with mastitis in Egypt [59]. The aac(6′)-Ib gene was not detected in any of our strains, even though it is frequently identified in ESBL *E. coli* strains of various hosts worldwide [21,60].

PMQR genes *qnrS*, *qnrB*, or both were identified in 5 out of the 11 fluoroquinolone resistant and in one fluoroquinolone susceptible ESBL-producing strains and were the gene family least frequently detected. Similarly, low PMQR detection rates have been reported in isolates obtained from cattle feces in Canada [61] and from lake water in Singapore [62]. The only study from Greece that has formerly identified concurrent presence of a *qnr* variant (*qnrS1*) with an ESBL gene (*bla*_CTX-M-15_) refers to a human *E. coli* isolate [63]. *qnrS* gene was detected in bovine strains and its co-occurrence with *bla*_CTX-M-15_ has previously been documented in *E. coli* of raw beef meat in Turkey [64]. The magpie’s isolate also presented the fluoroquinolone resistant phenotype due to carriage of *qnrS*. Our finding is in accordance with an earlier study from the Netherlands that reported coexistence of *qnrS* with *bla*_CTX-M-1_ in *E. coli* from a Northern lapwing and with *bla*_CTX-M-15_ in *E. coli* from a Black-headed gull [65]. Swine strains harbored *qnrS* and/or *qnrB*, as has previously been described for CTX-M-1group –producing *E. coli* strains isolated from fecal samples of pigs in China [66,67] and of piglets in India [68]. As for the six fluoroquinolone resistant strains that did not harbor PMQR genes, these probably expressed resistance due to mutations in the genes coding for DNA gyrase and topoisomerase IV [69]. In Greece, mutations in quinolone resistance-determining regions have been detected in ESBL *E. coli* strains of human origin that produced CTX-M-15 [35].

Trimethoprim/sulfamethoxazole resistance was mediated by the combined presence of *sul* and *dfrA* genes in all but one *E. coli* isolates. Sulfonamide resistance genes *sul1*, *sul2*, and *sul3* or different combinations of them were detected in the bovine and the swine strains. Braun et al. [60] also reported these resistance genes in ESBL *E. coli* isolates from feces of Egyptian dairy cattle. Notably, *sul3* is considered to be a rather rare sulfonamide resistance determinant [70]. In the magpie strain, the *sul1* and *sul2* genes were identified, as previously described for CTX-M-15 –producing strains of birds of prey in Germany and Mongolia [71] and of waterfowl in Pakistan [72]. Overall, the *sul2* gene presented the highest detection rate, which is consistent with former reports for isolates of humans, animals, and animal-derived foods [73]. Concerning trimethoprim resistance determinants, *dfrA1* predominated in cattle strains, whereas *dfrA1*, *dfrA7*, *dfrA17*, and *dfrA19* were evenly common in pig strains. Markedly, trimethoprim resistance genes were not detected in a bovine strain that presented reduced susceptibility to trimethoprim/sulfamethoxazole. This isolate only harbored *sul2*, which is associated with a trimethoprim/sulfamethoxazole susceptible phenotype, a fact implying the presence of an alternative resistance pathway in this strain.

Integrase genes were present in 13 of the livestock isolates as well as in the one from the magpie. Only class 1 integrons were detected, which are known to be the most common in enteric bacteria and are highly prevalent among isolates of pigs, cattle and wild birds [21,74,75]. In our study, *intI1* positive *E. coli* strains co-harbored *bla*_CTX-M-1/15_ and different combinations of resistance determinants for at least three classes of antibiotics. The presence of *intl1* could explain the resistance profiles of our strains, since inserted gene cassettes in these mobile genetic elements have been described to confer resistance to aminoglycosides, quinolones, trimethoprim, sulfonamides, and tetracyclines [76,77,78,79]. Finally, the ISE*cp1* element was detected in two bovine and four swine strains. It can be inferred that genes harbored by the ISE*cp1* positive strains are more likely to be widely disseminated, as this genetic platform has been associated with the mobilization and improved expression of *bla*_CTX-M_ [80,81]. In general, mobile genetic elements have contributed to the emergence of novel *E. coli* hybrid strains with distinct assortment of antimicrobial resistance traits [82].

Overall, multiple combinations of genes conferring antimicrobial resistance were detected in the ESBL-producing *E. coli* isolates of livestock origin. This finding could be attributed to the overuse or misuse of antimicrobials in animal husbandry [83] and is alarming since products derived from these animals are included in the daily human dietary [58]. Furthermore, MDR *E. coli* strains could potentially be transmitted from farmed animals to wildlife species or vice versa [84,85]. The magpie strain harbored resistance genes for all the tested antimicrobial classes, a fact that could be ascribed to antibiotic residues and ESBL-producing strains present in the environment due to human and livestock influence [86], as well as to the bird’s scavenging behavior. This resident wild bird lives in vicinity to humans and farmed animals and therefore could be contaminated by resistant bacteria from human or livestock offal as well as contribute to the spread of multidrug resistant ESBL bacteria. Thus, our results support previous studies that proposed wild birds as sentinels for antimicrobial resistance, reflecting the impact of human activities on the environment and highlight their possible role in the dissemination of multidrug resistant strains [87].

## 4. Materials and Methods

### 4.1. Study Design

In the context of an ongoing survey about β-lactamase producing Enterobacteriaceae of animal origin in Greece, 19 *E. coli* isolates that presented phenotypic resistance to third and fourth generation cephalosporins as well as a resistant phenotype to at least one class of non β-lactam antibiotics, were selected for further molecular characterization of resistance genes. All isolates were retrieved from non-duplicated fecal samples of clinically healthy animals using a sterile cotton swab (Transwab^®^ Amies, UK). Seven isolates were obtained from seven cattle, 11 from seven pigs and one from a Eurasian magpie. The wild bird isolate was retrieved after testing a total of 83 samples derived from 19 different wild bird species (Supplementary Materials File S2).

### 4.2. Isolation, Identification, and Antimicrobial Resistance Phenotype of ESBL-Producing Ε. coli

Swabs were directly streaked on ESBL selective media (CHROMID^®^ ESBL, BioMérieux, Marcy l’Etoile, France) and the plates were incubated aerobically at 37 °C for 24–48 h. Morphologically different colonies of pink to burgundy coloration, corresponding to *E. coli* growth, were sub-cultured on MacConkey agar. Identification and antimicrobial susceptibility testing of the isolates were performed using the Vitek-2 system (BioMérieux, Marcy l’Etoile, France), according to the manufacturer’s instructions. The AST-GN96 card was used in order to determine the minimum inhibitory concentration (MIC) of the following antimicrobials: ampicillin, amoxicillin/clavulanic acid, ticarcillin/clavulanic acid, cefalexin, cefalotin, cefoperazone, ceftiofur, cefquinome, imipenem, gentamicin, neomycin, flumequine, enrofloxacin, marbofloxacin, tetracycline, florfenicol, polymyxin B, and trimethoprim/sulfamethoxazole. Results were interpreted automatically by the Vitek-2 software, according to CLSI or CA-SFM criteria.

### 4.3. Phenotypic Confirmation of ESBL Production

ESBL production was phenotypically confirmed by the double disk synergy test, according to EUCAST guidelines [88]. Antibiotic disks containing cefotaxime (30 μg), ceftazidime (30 μg), cefepime (30 μg), and amoxicillin/clavulanate acid (20 μg/10 μg) were applied at a distance of 20 mm (center to center) on Mueller Hinton agar that was pre-inoculated with an 0.5 McFarland inoculum. Following incubation, any enhanced zone of inhibition between cephalosporin disks and the amoxicillin/clavulanic acid disk or a ‘keyhole’ formation in the direction of the disk containing clavulanic acid were considered as evidence for the presence of an ESBL producing strain. In cases of ambiguous results, a combination disk test was applied, using cefotaxime and ceftazidime disks (30 μg each), alone and in combination with clavulanic acid (10 μg). A difference of ≥5 mm in zone diameter among single and combined with clavulanic acid antimicrobial agents was interpreted as ESBL production.

### 4.4. Molecular Genotyping of the ESBL-Producing E. coli

The CarbDetect AS-2 Kit (Abbott, Jena, Germany) was used, according to the manufacturer’s instructions, to detect the AMR genotype. This microarray kit simultaneously detects a total of 134 genes, as presented by Braun et al. [89] and in Supplementary Materials File S3. The “result collector” software, provided by Abbott, automatically summarized the data. An antibiotic resistance genotype was defined as a group of genes, which have been described to confer resistance to a family of antibiotics (e.g., the genotype “*bla*_CTX-M1/15_, *bla*_TEM_” confers resistance to third generation cephalosporins).

For strain S7-1, the Nanopore Oxford MinION platform was used to sequence the whole genome, in order to prove the presence or absence of a microarray detected carbapenemase gene, *bla*_OXA-134-like_. Briefly, size selection was performed using AMPure beads in a ratio 1:1 (v/v) with the isolated DNA sample. The DNA library was generated using the nanopore sequencing kit SQK-LSK109 (Oxford Nanopore Technologies, Oxford, UK), according to manufacturer’s instructions. The used Flongle flow cell FLO-FLG001 (R9.4.1) was primed by the flow cell priming kit EXP-FLP002 (Oxford Nanopore, Oxford, UK). The protocol named “Genomic DNA by Ligation” was used in version GDE_9063_v109_revW_14AAug2019 (Last update: 9 December 2020). The guppy basecaller (v4.4.2., Oxford Nanopore Technologies, Oxford, UK) translated and trimmed the MinION raw data (fast5) into quality tagged sequence reads (4000 reads per fastq-file). Flye (v2.8.3) was used to assemble all reads to two large contigs (the chromosome and one plasmid). Then, a racon-medaka (racon v1.4.3; medaka v1.2.0) pipeline was applied for polishing. The tool Abricate (v1.1.0) was used to identify possible resistance genes in both chromosome and plasmid (Last update: 19 April 2020) [90].

## 5. Conclusions

Our study presented the antimicrobial resistance profile of ESBL-producing *E. coli* strains isolated from cattle, pigs, and a wild bird in Greece. All the strains that were selected for analysis harbored *bla*_CTX-M-1/15_ along with various other genes conferring resistance to six classes of antimicrobials. This finding underlines the wide dissemination of multidrug resistant bacteria in diverse ecosystems and emphasizes the need for an integrated antimicrobial surveillance system. Further studies are required to fully illustrate the occurrence of MDR ESBL-producing isolates, investigate their origin and unravel the dynamics of their transmission in Greece.

## Figures and Tables

**Table 1:** Antimicrobial resistance phenotype and genotype of the ESBL-producing *E. coli* isolates.

Isolate ^1^	Antimicrobial Resistance Phenotype	Antimicrobial Resistance Genotype						
		β-lactamases genes	Aminoglycoside Resistance Genes	PMQRGenes	Sulfonamide Resistance Genes	Trimethoprim Resistance Genes	Macrolide Resistance Genes	Genes Associated with Mobile Genetic Elements
B1	AMP, AMC, TCC, CEX, CF, CFP, CEF, CEQ, GEN, NEO, FLU, ENR, MRB, TET, SXT	*bla*_CTX-M1/15_, *bla*_TEM_	*aadA1, aphA*	*-*	*sul1*	*dfrA1*	*-*	*intI1*
B2	AMP, AMC, TCC *, CEX, CF, CFP, CEF, CEQ, GEN, NEO, FLU, TET, SXT	*bla*_CTX-M1/15_ *, bla*_TEM_	*aadA1, aphA, strA, strB*	*-*	*sul1, sul2*	*dfrA1*	*mph*	*intI1*
B3	AMP, CEX, CF, CFP, CEF, CEQ, GEN, NEO *, TET, SXT	*bla*_CTX-M1/15_, *bla*_TEM_	*aadA1, aadA2, aphA, strA, strB*	*-*	*sul1, sul2*	*dfrA1*	*-*	*intI1*
B4	AMP, CEX, CF, CFP, CEF, CEQ, GEN, NEO *, FLU, TET, SXT	*bla*_CTX-M1/15_, *bla*_TEM_, *bla*_MIR_	*aadA1, aadA2, aphA, strA, strB*	*-*	*sul1, sul2, sul3*	*dfrA1*	*-*	*intI1*
B5	AMP, CEX, CF, CFP, CEF, CEQ, NEO, TET	*bla*_CTX-M1/15_, *bla*_TEM_	*aphA, strA, strB*	-	*sul2*	-	-	tnpIS*Ecp*1
B6	AMP, CEX, CF, CFP, CEF, CEQ, GEN, NEO, TET, SXT	*bla*_CTX-M1/15_, *bla*_TEM_	*aadA1, aadA2, aphA, strA, strB*	*-*	*sul1, sul2*	*dfrA1, dfrA5*	*-*	*intI1*
B7	AMP, AMC, CEX, CF, CFP, CEF, CEQ, NEO *, FLU *, ENR *, TET, SXT	*bla*_CTX-M1/15_, *bla*_TEM_	*aphA, strA, strB*	*qnrS*	*sul2*	-	-	tnpIS*Ecp*1
S1	AMP, CEX, CF, CFP, CEF, CEQ, GEN, NEO *, FLU, ENR, MRB, TET	*bla*_CTX-M1/15_, *bla*_TEM_	*aadA1, aphA,*	-	*sul2, sul3*	-	-	tnpIS*Ecp*1
S2	AMP, CEX, CF, CFP, CEF, CEQ, TET, SXT	*bla*_CTX-M1/15_	*aadA1, aadA4*	*-*	*sul1, sul2*	*dfrA7, dfrA17, dfrA19*	*-*	*intI1,* tnpIS*Ecp*1
S3-1	AMP, AMC, TCC *, CEX, CF, CFP, CEF, CEQ, GEN, FLU *, ENR *, TET, SXT	*bla*_CTX-M1/15_	*aadA1, aadA4*	*qnrB*	*sul1*	*dfrA1, dfrA7, dfrA17, dfrA19*	*mph, mrx*	*intI1*
S3-2	AMP, AMC, TCC *, CEX, CF, CEF, CEQ, TET, SXT	*bla*_CTX-M1/15_, *bla*_TEM_	*aadA1, strA, strB*	*-*	*sul1, sul2*	*dfrA1, dfrA14, dfrA15*	*mph, mrx*	*intI1*
S3-3	AMP, AMC, TCC, CEX, CF, CFP, CEF, CEQ, GEN, FLU, ENR *, TET, SXT	*bla*_CTX-M1/15_, *bla*_TEM_	*aadA1, aadA2, aadA4, strA, strB*	*qnrB, qnrS*	*sul1, sul2*	*dfrA1, dfrA7, dfrA12, dfrA17, dfrA19*	*mph, mrx*	*intI1*
S4-1	AMP, CEX, CF, CFP, CEF, CEQ, TET, SXT	*bla*_CTX-M1/15_	*aadA1, aadA2*	*-*	*sul1, sul2*	*dfrA12*	*-*	*intI1*
S4-2	AMP, CEX, CF, CFP, CEF, CEQ, TET, SXT	*bla*_CTX-M1/15_, *bla*_TEM_	*aadA1, aadA2, aadA4*	*-*	*sul1, sul2, sul3*	*dfrA1, dfrA7, dfrA12, dfrA15, dfrA17, dfrA19*	*-*	*intI1,* tnpIS*Ecp*1
S5	AMP, CEX, CF, CFP, CEF, CEQ, NEO *, FLU, ENR, MRB, TET, SXT	*bla*_CTX-M1/15_, *bla*_TEM_	*aphA, strA, strB*	*-*	*sul2*	*dfrA5*	*-*	*intI1*
S6	AMP, AMC, TCC *, CEX, CF, CFP, CEF, CEQ, GEN, NEO *, FLU, ENR, MRB, TET, SXT	*bla*_CTX-M1/15_, *bla*_TEM_	*aadA1, aadA2, aadA4, aphA, strA, strB*	*-*	*sul1, sul2,* *sul3*	*dfrA1, dfrA7, dfrA12, dfrA14, dfrA17, dfrA19*	*mph, mrx*	*intI1,* tnpIS*Ecp*1
S7-1	AMP, CEX, CF, CFP, CEF, CEQ, FLU *, ENR *	*bla*_CTX-M1/15_	*aadA2*	*qnrS*	-	-	*mph, mrx*	-
S7-2	AMP, CEX, CF, CFP, CEF, CEQ, TET	*bla*_CTX-M1/15_	*aadA2*	*qnrS*	-	-	*mph, mrx*	-
WB1	AMP, CEX, CF, CFP, CEF, CEQ, FLU, ENR *, TET, SXT	*bla*_CTX-M-1/15_	*aadA4, strA, strB*	*qnrS*	*sul1, sul2*	*dfrA7, dfrA17, dfrA19*	*mph, mrx*	*intI1*

^1^ B—bovine strains; S—swine strains; WB—Eurasian magpie strain; AMP—ampicillin; AMC—amoxicillin/clavulanic acid; TCC—ticarcillin/clavulanic acid; CEX—cefalexin; CF—cefalotin; CFP—cefoperazone; CEF—ceftiofur; CEQ—cefquinome; GEN—gentamicin; NEO—neomycin; FLU—flumequine; ENR—enrofloxacin; MRB—marbofloxacin; TET—tetracycline; SXT—trimethoprim/sulfamethoxazole; * intermediate resistance; - the isolate did not harbor genes of this category.

## Data Availability

Most data for this study are presented in the Supplementary Files. The remaining data are available on request from the corresponding author. The data are not publicly available as they are part of the PhD thesis of the first author, which has not yet been examined, approved, and uploaded in the official depository of PhD theses from Greek Universities.

## Supplementary Materials

The following are available online at https://www.mdpi.com/article/10.3390/antibiotics10040389/s1, Supplementary Materials File S1: Resistance genes and their locations identified using Whole Genome Sequencing of the strain S7-1, Supplementary Materials File S2: Wild bird species included in the study, number of samples per species and number of ESBL-producing Escherichia coli isolates obtained per species, Supplementary Materials File S3: Genes Detected by the CarbDetect AS-2 Kit.
