# Supplementary material for: Antimicrobial Resistance Genes in ESBL-Producing Escherichia coli Isolates from Animals in Greece
Source: Antibiotics (Basel). 2021 Apr 4;10(4):389. doi: 10.3390/antibiotics10040389 (PMC8067336; doi:10.3390/antibiotics10040389)
Supplement: Supplementary file 1 [file antibiotics-10-00389-s001.zip › Supplementary File S2. Wild Bird Species Sampled.docx]

| **Animal Species** | **Number of Samples** | **Number of ESBL-producing *Escherichia coli*** |
| --- | --- | --- |
| Golden pheasant  (*Chrysolophus pictus*) | 2 | 0 |
| Common pheasant  (*Phasianus colchicus*) | 7 | 0 |
| Rock partridge  (*Alectoris graeca*) | 3 | 0 |
| Eurasian woodcock  (*Scolopax rusticola*) | 11 | 0 |
| Long-eared owl  (*Asio otus*) | 2 | 0 |
| Eurasian eagle-owl  (*Bubo bubo*) | 3 | 0 |
| House sparrow  (*Passer domesticus*) | 5 | 0 |
| Common buzzard  (*Buteo buteo*) | 5 | 0 |
| Lesser kestrel  (*Falco naumanni*) | 1 | 0 |
| Little owl  (*Athene noctua*) | 1 | 0 |
| Common starling  (*Sturnus vulgaris*) | 9 | 0 |
| Common wood pigeon (*Columba palumbus*) | 3 | 0 |
| Song thrush  (*Turdus philomelos*) | 14 | 0 |
| Eurasian Magpie  (*Pica pica*) | 3 | 1 |
| European goldfinch  (*Carduelis carduelis*) | 5 | 0 |
| Common blackbird  (*Turdus merula*) | 3 | 0 |
| Domestic Muscovy duck  (*Cairina moschata domestica*) | 1 | 0 |
| Mallard  (*Anas platyrhynchos*) | 3 | 0 |
| Domestic goose  (*Anser cygnoides domesticus*) | 1 | 0 |
| TOTAL | 83 | 1 |

Supplementary File S2: Wild bird species included in the study, number of samples per species and number of ESBL-producing *Escherichia coli* isolates obtained per species.
