# Supplementary material for: Antimicrobial Resistance Genes in ESBL-Producing Escherichia coli Isolates from Animals in Greece
Source: Antibiotics (Basel). 2021 Apr 4;10(4):389. doi: 10.3390/antibiotics10040389 (PMC8067336; doi:10.3390/antibiotics10040389)
Supplement: Supplementary file 1 [file antibiotics-10-00389-s001.zip › Supplementary File S3. Genes Detected by the CarbDetect AS-2 Kit.docx]

| **Supplementary File S3. Genes Detected by the CarbDetect AS-2 Kit** | | |
| --- | --- | --- |
| **Target genes** | **Gene function** | **Reference**  **sequence** |
| basC | acinetobactin biosynthesis protein of Acinetobacter baumannii | AY571146 |
| cfa | colicin five activity protein of *Citrobacter freundii* and *Citrobacter braakii* | U09771 |
| dnaE | DNA polymerase III subunit alpha | U00096.3 |
| ecfX | extracytoplasmic function sigma factor of *Pseudomonas aeruginosa* [LavenirR-JocktaneD-2007] | DQ996558 |
| efp | elongation factor P of *Acinetobacter baumannii* | CP001172 |
| gad | glutamate decarboxylase of *Escherichia coli* | AE014075 |
| ihfA | integration host factor subunit alpha | U00096.3 |
| invA | invasin A, highly specific for genus *Salmonella* | CP000026 |
| ipaH9.8 | invasion plasmid antigen | AF047365 |
| khe | klebsolysin of *Klebsiella pneumoniae* | AF293352 |
| lacY | lactose permease; the lacY gene is missing in all *Shigella spp.* | U00096 |
| pld | phospholipase D of *Acinetobacter baumannii* | CP000521 |
| rrs | 16S rRNA | U00096.3 |
| blaBIC | carbapenemase, class A beta-lactamase | GQ260093 |
| blaDIM | carbapenemase, class B metallo beta-lactamase | KC004136 |
| blaGES | carbapenemase, class A beta-lactamase | AY219651 |
| blaGIM | carbapenemase, class B metallo beta-lactamase | consensus |
| blaGOB | carbapenemase, class B metallo beta-lactamase | consensus |
| blaIMI-3 (NmcA) | carbapenemase, class A beta-lactamase associated with imipenem resistance | AY780889 |
| blaIMI-R | regulator of blaIMI-3 (NMC-A) | Z21956 |
| blaIMP | carbapenemase, class B metallo beta-lactamase | consensus |
| blaIMP25 (blaSIM-1) | carbapenemase, class B metallo beta-lactamase (synonym: blaSIM) | EU686387 |
| blaIMP35 | carbapenemase, class B metallo beta-lactamase | JQ432564 |
| blaIND | carbapenemase, class B metallo beta-lactamase of Chryseobacterium | consensus |
| blaKHM | carbapenemase, class B metallo beta-lactamase | consensus |
| blaKPC | carbepenemase, class A serin beta-lactamase | consensus |
| blaNDM | carbapenemase, class B metallo beta-lactamase (New Dehli metallo beta-lactamase) | consensus |
| blaPAM-1 | carbapenemase, subclass B3 metallo beta-lactamase (*Pseudomonas alcaligenes* metallo-betalactamase) | AB858498 |
| blaSFH-1 | carbapenemase, class B metallo beta-lactamase | AF197943 |
| blaSMB-1 | carbapenemase, class B metallo beta-lactamase (*Serratia marcescens*) | AB636283 |
| blaSME | carbapenemase, class A beta-lactamase (*Serratia marcescens*) | consensus |
| blaSPM-1 | carbapenemase, class B metallo beta-lactamase | AY341249 |
| blaTMB-1 | carbapenemase, class B metallo beta-lactamase | FR771847 |
| blaVIM | carbapenemase, class B metallo beta-lactamase | Consensus |
| blaVIM-2 | carbapenemase, class B metallo beta-lactamase | AF191564 |
| blaVIM-7 | carbapenemase, class B metallo beta-lactamase | AJ536835 |
| blaOXA-23-like | carbapenemase, class D beta-lactamase | AJ132105 |
| blaOXA-40-like | carbapenemase, class D beta-lactamase | AF509241 |
| blaOXA-48-like | carbapenemase, class D beta-lactamase | AY236073 |
| blaOXA-51-like | carbapenemase, class D beta-lactamase | CP000863 |
| ISABa1 to blaOXA-51 | Insertion sequence ABa1 is adjacent to blaOXA-51-like gene. This combination mediated carbapenem resistant in Acinetobacter baumannii isolates (Turton et al. 2006). | CP001921 |
| no ISABa1 to blaOXA-51 | Insertion sequence ABa1 is not adjacent to blaOXA-51-like gene. This combination does not mediate carbapenem resistant in Acinetobacter baumannii isolates (Turton et al. 2006). | CU459141 |
| blaOXA-54 | carbapenemase, class D beta-lactamase | AY500137 |
| blaOXA-55 | carbapenemase, class D beta-lactamase | AY343493 |
| blaOXA-58 | carbapenemase, class D beta-lactamase | AY665723 |
| blaOXA-134/235/284 | carbapenemase blaOXA-134-like, class D beta-lactamase | AYHO01000005 |
| blaOXA-143/182/253/255 | carbapenemase blaOXA-40-like, class D beta-lactamase | GQ861437 |
| blaOXA-181/232 | carbapenemase blaOXA-48-like, class D beta-lactamase | CP000469 |
| blaOXA-214 | carbapenemase, class D beta-lactamase | JN861783 |
| blaOXA-279 | carbapenemase, class D beta-lactamase | APOK01000044 |
| blaOXA-292 | carbapenemase, class D beta-lactamase | APRH01000012 |
| blaCME | extended spectrum beta-lactamase, class A | AF033200 |
| blaCTX-M1/15 | extended spectrum beta-lactamase, class A | X92506, HQ202266 |
| blaCTX-M2 | extended spectrum beta-lactamase, class A | AF286192 |
| blaCTX-M8 | extended spectrum beta-lactamase, class A | AY750914 |
| blaCTX-M9 | extended spectrum beta-lactamase, class A | FQ482074 |
| blaMIR | extended spectrum beta-lactamase, class C | M37839 |
| blaMOX-CMY9 | extended-spectrum beta-lactamase precursor, class C | AF381617 |
| blaPER-1 | extended-spectrum beta-lactamase, class A beta-lactamase PER-1 | Z21957 |
| blaPER-2 | extended-spectrum beta-lactamase, class A beta-lactamase PER-2 | X93314 |
| blaSHV | class A beta-lactamase - consensus probe for blaSHV genes, including extended-spectrum beta-lactamases | consensus |
| blaTEM | class A beta-lactamase - consensus probe for blaTEM genes, including extended-spectrum beta-lactamases | consensus |
| blaVEB | extended-spectrum beta-lactamase, class A | consensus |
| blaOXA-18 | extended spectrum beta-lactamase, class D | EU503121 |
| blaOXA-45 | extended spectrum beta-lactamase, class D | AJ519683 |
| blaOXA-1 | narrow spectrum beta-lactamase, class D | AY458016 |
| blaOXA-7 | narrow spectrum beta-lactamase, class D | AY866525 |
| blaOXA-9 | narrow spectrum beta-lactamase, class D | M55547 |
| blaOXA-2 | consensus probe for extended and narrow spectrum class D beta-lactamases belonging to group blaOXA-2 | consensus |
| blaOXA-10 | consensus probe for extended and narrow spectrum class D beta-lactamases belonging to group blaOXA-10 | consensus |
| blaOXA-60 | narrow spectrum beta-lactamase, class D | AF525303 |
| blaACC | AmpC beta-lactamase | EF554600 |
| blaACT | AmpC beta-lactamase | U58495 |
| blaCMY | AmpC beta-lactamase | consensus |
| blaDHA | AmpC beta-lactamase | EF406115 |
| blaFOX | AmpC beta-lactamase | consensus |
| blaMOX | AmpC beta-lactamase | consensus |
| aac(3') | 3-N-aminoglycoside acetyltransferase; associated with resistance to astromicin; gentamicin; sisomicin (consensus) | consensus |
| aac(3')-Ia | 3-N-aminoglycoside acetyltransferase; associated with resistance to astromicin; gentamicin; sisomicin | U90945 |
| aac(3')-Ib | 3-N-aminoglycoside acetyltransferase; associated with resistance to astromicin; gentamicin; sisomicin | KJ679408 |
| aac(3')-Ic | 3-N-aminoglycoside acetyltransferase; associated with resistance to astromicin; gentamicin; sisomicin | AJ511268 |
| aac(3')-Ie | 3-N-aminoglycoside acetyltransferase; associated with resistance to astromicin; gentamicin; sisomicin | AY458224 |
| aac(3')-IVa | 3-N-aminoglycoside acetyltransferase; associated with resistance to apramycin; dibekacin; gentamicin; netilmicin; sisomicin; tobramycin | EU784152 |
| aac(6') | aminoglycoside 6'-N-acetyltransferase, associated with resistance to amikacin; dibekacin; isepamicin; netilmicin; sisomicin; tobramycin (consensus) | consensus |
| aac(6')-31 | aminoglycoside 6'-N-acetyltransferase; associated with resistance to streptomycin, spectinomycin | AJ640197 |
| aac(6')-Ib | aminoglycoside 6'-N-acetyltransferase; associated with resistance to streptomycin, spectinomycin | M21682 |
| aac(6')-II | aminoglycoside 6'-N-acetyltransferase; associated with resistance to streptomycin, spectinomycin | EF127959 |
| aac(6')-IIa | aminoglycoside 6'-N-acetyltransferase; associated with resistance to streptomycin, spectinomycin | EU912537 |
| aac(6')-IIc | aminoglycoside 6'-N-acetyltransferase; associated with resistance to streptomycin, spectinomycin | EU855788 |
| aac-aph | 6'-aminoglycoside-N-acetyltransferase/2''-aminoglycoside phosphotransferase; associated with resistance to gentamycin | AE017171 |
| aadA1 | aminoglycoside adenyltransferase; associated with resistance to streptomycin, spectinomycin | EU704128 |
| aadA2 | aminoglycoside adenyltransferase; associated with resistance to streptomycin, spectinomycin | EU704128 |
| aadA4 | aminoglycoside adenyltransferase; associated with resistance to streptomycin, spectinomycin | Z50802.3 |
| aadB | 2''-aminoglycoside nucleotidyltransferase | L06418.4 |
| ant2 | aminoglycoside (2'') adenylyltransferase; associated with resistance to dibekacin; gentamicin; kanamycin; sisomicin; tobramycin | L06418.4 |
| aphA | aminoglycoside 3'-phosphotransferase; kanamycin resistance protein | AY260546.3 |
| armA | 16S rRNA methylase, associated with aminoglycoside resistance | AB117519 |
| grm | 16S rRNA methylase, associated with gentamicin resistance | M55521 |
| npmA | 16S rRNA methylase, associated with aminoglycoside resistance | AB261016 |
| rmtA | 16S rRNA methylase, associated with aminoglycoside resistance | AB083212 |
| rmtB | 16S rRNA methylase, associated with aminoglycoside resistance | DQ345788 |
| rmtC | 16S rRNA methylase, associated with aminoglycoside resistance | AB194779 |
| rmtD | 16S rRNA methylase, associated with aminoglycoside resistance | DQ914960 |
| strA | aminoglycoside-3''-phosphotransferase (locus A); associated with resistance to streptomycin | EF090911 |
| strB | aminoglycoside-6''-phosphotransferase; associated with resistance to streptomycin | EF090911 |
| mph | macrolide 2'-phosphotransferase | consensus |
| mrx | member of macrolide inactivation gene cluster mphA-mrx-mphR | consensus |
| qepA | QepA - fluoroquinolone/quinolone efflux pump | AM886293 |
| qnrA1 | quinolone or fluoroquinolone resistance protein | AY931018 |
| qnrB | quinolone or fluoroquinolone resistance protein | AB281054 |
| qnrC | quinolone or fluoroquinolone resistance protein | EU917444 |
| qnrD | quinolone or fluoroquinolone resistance protein | FJ228229 |
| qnrS | quinolone or fluoroquinolone resistance protein | AM234722 |
| sul1 | dihydropteroate synthetase type 1 | AJ698325 |
| sul2 | dihydropteroate synthetase type 2 | DQ464881 |
| sul3 | dihydropteroate synthetase type 3 | AJ459418 |
| dfrA1 | dihydrofolate reductase type 1 | AJ884723 |
| dfrA12 | dihydrofolate reductase type 12 | AB154407 |
| dfrA13 | dihydrofolate reductase type 13 (synonym A21) | Z50802.3 |
| dfrA14 | dihydrofolate reductase type 14 | AJ313522 |
| dfrA15 | dihydrofolate reductase type 15 | Z83311 |
| dfrA17 | dihydrofolate reductase type 17 | AF169041 |
| dfrA19 | dihydrofolate reductase type 19 | AJ310778 |
| dfrA5 | dihydrofolate reductase type 5 | AB188269 |
| dfrA7 | dihydrofolate reductase type 7 | AB161450 |
| intI1 | class 1 integron integrase | AY260546.3 |
| intI2 | class 2 integron integrase | AY183453 |
| intI3 | class 3 integron integrase | EF469602 |
| tnpISEcp1 | transposase for the transposon ISEcp1 | AB543698 |
| oqxA | OqxA - membran fusion protein, component of RND-type multidrug efflux pump, associated with olaquindox resistance | EU370913 |
| oqxB | OqxB - integral membrane protein, component of RND-type multidrug efflux pump, associated with olaquindox resistance | EU370913 |
| higA | higA is the antitoxin of the translation-dependent mRNA interferase toxin higB | U43847 |
| higB | Ectopic expression of higB causes inhibition of cell growth which is alleviated by co-expression of higA | U43847 |
| splA | splA is the antitoxin of the translation-dependent mRNA interferase toxin splT | EU294228 |
| splT | Ectopic expression of splT causes inhibition of cell growth which is alleviated by co-expression of splA | EU294228 |
